# Quantitative disease resistance in wild *Silene vulgaris* to its endemic pathogen *Microbotryum silenes‐inflatae*

**DOI:** 10.1002/ece3.10797

**Published:** 2023-12-20

**Authors:** Michael E. Hood, Sydney Nelson, Jae‐Hoon Cho, Michelle Launi, Janis Antonovics, Emily L. Bruns

**Affiliations:** ^1^ Department of Biology Amherst College Amherst Massachusetts USA; ^2^ Department of Biology University of Virginia Charlottesville Virginia USA; ^3^ Department of Biology University of Maryland at College Park College Park Maryland USA

**Keywords:** anther smut, disease resistance, *Microbotryum*, natural variation, quantitative resistance, *Silene*

## Abstract

The evolution of disease resistances is an expected feature of plant–pathogen systems, but whether the genetics of this trait most often produces qualitative or quantitative phenotypic variation is a significant gap in our understanding of natural populations. These two forms of resistance variation are often associated with differences in number of underlying loci, the specificities of host–pathogen coevolution, as well as contrasting mechanisms of preventing or slowing the infection process. Anther‐smut disease is a commonly studied model for disease of wild species, where infection has severe fitness impacts, and prior studies have suggested resistance variation in several host species. However, because the outcome of exposing the individual host to this pathogen is binary (healthy or diseased), resistance has been previously measured at the family level, as the proportion of siblings that become diseased. This leaves uncertain whether among‐family variation reflects contrasting ratios of segregating discrete phenotypes or continuous trait variation among individuals. In the host *Silene vulgaris*, plants were replicated by vegetative propagation in order to quantify the infection rates of the individual genotype with the endemic anther‐smut pathogen, *Microbotryum silenes‐inflatae*. The variance among field‐collected families for disease resistance was significant, while there was unimodal continuous variation in resistance among genotypes. Using crosses between genotypes within ranked resistance quartiles, the offspring infection rate was predicted by the parental resistance values. While the potential remains in this system for resistance genes having major effects, as there were suggestions of such qualitative resistance in a prior study, here the quantitative disease resistance to the endemic anther‐smut pathogen is indicated for *S. vulgaris*. The variation in natural populations and strong heritability of the trait, combined with severe fitness consequences of anther‐smut disease, suggests that resistance in these host populations is highly capable of responding to disease‐induced selection.

## Graphical abstract

Infectious disease is a ubiquitous component of wildlife biology, but how disease resistance varies among individuals is a significant gap in our understanding of natural populations. Using a disease with severe fitness consequences for a wild plants species, we reveal resistance to display continuous phenotypic variation with a high degree of heritability, indicating that the host populations are highly capable of responding to disease‐induced selection.

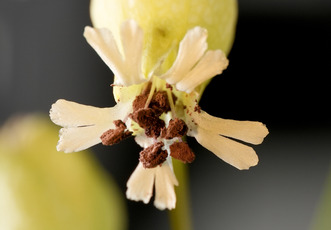

## 1 INTRODUCTION

The factors determining host vulnerability to infection are central to disease ecology, affecting pathogen distributions, epidemic spread, and disease impacts on agriculture or conservation efforts (Burdon & Thrall, 2014). Evolutionary change is also critically important when the risk of infection has a strong heritable component, and details of the underlying genetics then determine the outcome and dynamics of host–pathogen coevolution (Buckingham & Ashby, 2022; Thrall et al., 2016). Although heritable variation in resistance is of great utility in efforts for breeding resistance in agricultural crops, our understanding of wild plant populations is much more limited (Burdon & Thrall, 2009; Chappell & Rausher, 2011; Demirjian et al., 2023; Kahlon et al., 2021). This knowledge gap restricts our ability to predict how diseases influence natural systems, including pathogen persistence, virulence evolution, and host range (Gandon & Michalakis, 2000; Gates et al., 2018), as well as our ability to mitigate risks of diseases that emerge into domestic populations (Alexander, 1992; Burdon & Thrall, 2014; Jones, 2001; Zhang & Batley, 2020).

Heritable resistance variation in plants is typically viewed as having two main forms (Corwin & Kliebenstein, 2017; Kushalappa et al., 2016; Poland et al., 2009). The first includes mechanisms expected to prevent infection by the pathogen, operating in a strain‐specific manner and where response to that pathogen is inherited as discrete variation at a very small number of the genes (i.e., qualitative resistance, or in particular, gene‐for‐gene resistance). The second involves less specific mechanisms that decrease the progress of infection or reduce pathogen reproduction and are inherited as continuous variation through the combined effects of multiple genes (i.e., quantitative resistance, sensu Falconer, 1986, Simms & Rausher, 1992). Parallels to both qualitative host genotype‐by‐pathogen genotype interactions and quantitative resistance occur in animals, including in humans (Råberg, 2023; Sternberg et al., 2013; Wilfert & Schmid‐Hempel, 2008). In these contexts, qualitative and quantitative can refer to both variation in the phenotypic outcome of attempted infection and the pattern of inheritance of the resistance trait (Niks et al., 2015). Both forms of resistance may exist in the same host–pathogen system and even co‐occur within the same host population (Ericson & Burdon, 2009; Laine et al., 2011; Susi & Laine, 2015), and there are likely to be implications for how each form of resistance affects selection on the other (Burdon, 1997; Hulse et al., 2023; Miller & Metcalf, 2019; Price et al., 2004). Still, the distinction between qualitative and quantitative resistance is not absolute, with studies showing that the network of resistance genes involved can overlap (French et al., 2016), and there are exceptions to the generalized patterns noted above (Niks et al., 2015). Moreover, understanding the balance between qualitative and quantitative resistance in nature is identified as a persistent challenge, with the need for investigations on a broader range of study systems (Buckingham & Ashby, 2022; Burdon & Thrall, 2009; Chappell & Rausher, 2011; Delplace et al., 2020; Demirjian et al., 2023; Laine et al., 2011).

**FIGURE 1:**
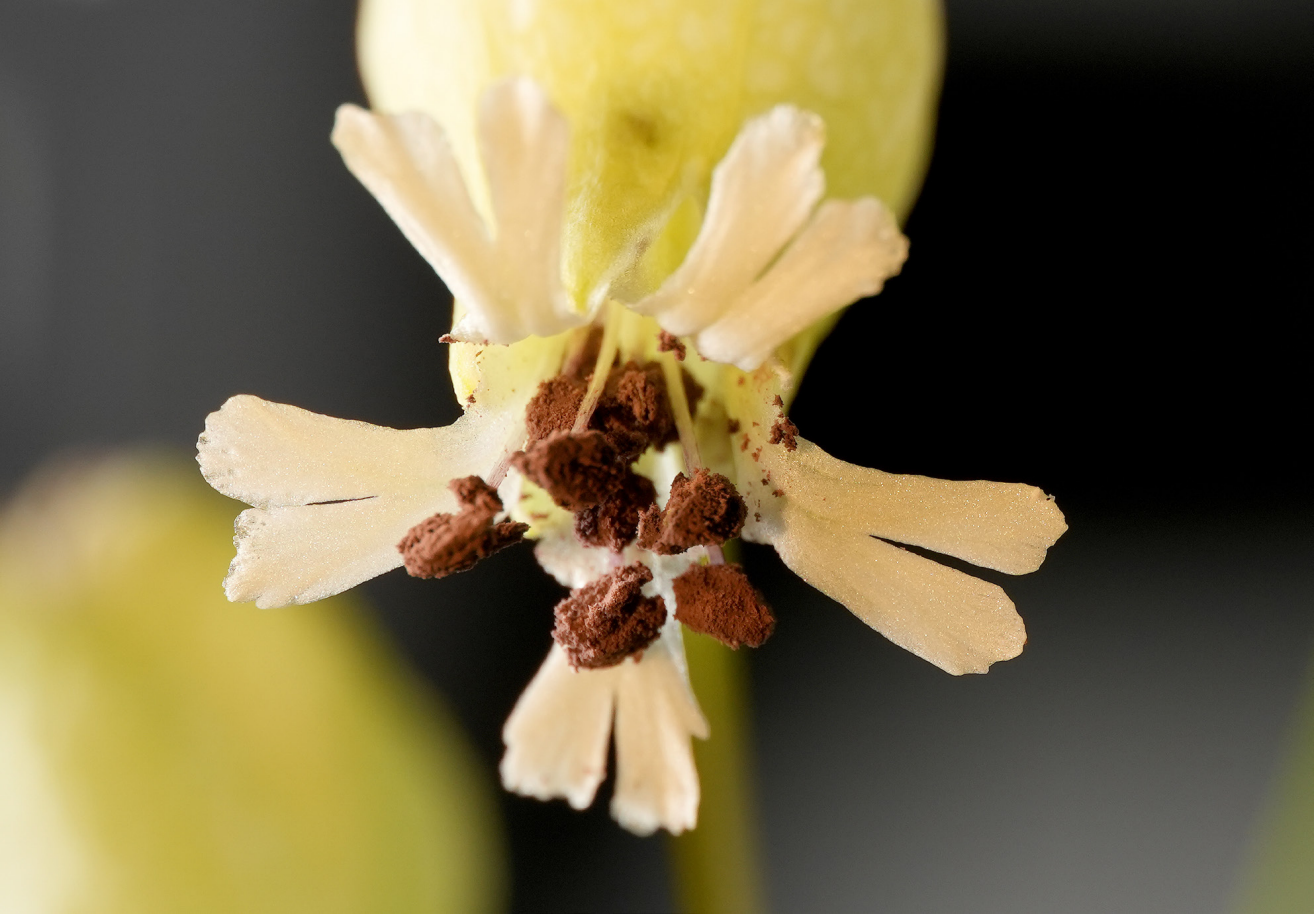
Anther‐smut disease of *Silene vulgaris*, caused by the fungus *Microbotryum silenes‐inflatae*. The fungus replaces the pollen of the anthers with masses of purple‐brown spores, which can be dispersed by visiting pollinators.

Anther‐smut disease has been an important model for many aspects of disease in wild species (Antonovics et al., 2002; Bernasconi et al., 2009), and the combination of frequent high disease prevalence and severe fitness impacts make anther‐smut disease a system in which strong selective pressure for resistance is expected (Bruns et al., 2015, 2019). Affecting mostly plants in the Caryophyllaceae, anther smut is caused by host‐specific fungi in the genus *Microbotryum*, and the systemic infections result in sterility of the host through the replacement of pollen with fungal spores and prevention of ovary development (Figure 1; Schäfer et al., 2010). However, studying resistance inheritance by the traditional use of inbred host lines is generally not feasible due to inbreeding depression in the host species (Emery & McCauley, 2002; Teixeira et al., 2009). Moreover, as the disease is sterilizing, crosses involving susceptible infected plants are generally not possible. Lastly, due to the systemic nature of infection, the disease outcome for a particular host plant is generally assessed as a binary response (i.e., healthy or diseased; e.g., Arnaise et al., 2020; Cafuir et al., 2007; Ouborg et al., 2000; Roche et al., 1995) and resistance can only be further quantified at the host‐family level using the proportion of siblings that become diseased upon exposure to the pathogen (e.g., Bruns et al., 2022; Carlsson‐Granér, 1997; Carlsson‐Granér & Thrall, 2015; Chung et al., 2012; Kaltz & Shykoff, 2002; Lerner et al., 2021).

Despite these limitations, several studies have demonstrated variation in resistance to anther‐smut disease. In *Silene latifolia*, field‐collected host genotypes, each from a separate family, varied in their rates of infection by *Microbotryum lychnidis‐dioicae* (Alexander, 1989; Alexander et al., 1993). Those studies used experimentally produced plant clones to replicate each host genotype, thus enabling the measurement of individual‐level resistance, which then predicted the family‐level infection rates of their offspring (Alexander & Antonovics, 1995). A tissue culture approach to replicating host genotypes was used in *Silene vulgaris*, and while limited in sample size, that study suggested that host genotypes varied discretely and could be classified into high versus low levels of resistance (i.e., qualitative resistance variation; Cafuir et al., 2007). However, that study used a pathogen foreign to *S. vulgaris* (*M. lychnidis‐dioicae* from *S. latifolia* in a population where a host shift had recently occurred; Antonovics et al., 2002). Otherwise, the genetics of resistance in *S. vulgaris* to its naturally occurring pathogen, *M. silenes‐inflatae*, has not been investigated previously, other than showing there is a range of family‐level resistances (Lerner et al., 2021). Thus, it is an open question whether natural disease resistance to endemic anther smut is characterized by quantitative disease resistance or discretely varying qualitative resistance.

In the current study, we evaluated resistance variation and inheritance in *S. vulgaris* to its endemic anther‐smut pathogen, *Microbotryum silenes‐inflatae*. Our main goals were (1) to assess whether the healthy‐versus‐diseased outcome of inoculating particular plants reflects continuously or discretely varying resistance phenotypes, and (2) to determine whether heritability of the individual variation can be confirmed. A vegetative propagation approach was used to replicate plants and then measure individual‐level resistance of *S. vulgaris* genotypes that were collected from the field. These plants, in turn, served as selected parents for an offspring generation that was also assessed for resistance. Even though anther smut is a disease where infection of the particular individual has a binary outcome, quantitative variation in the probability of becoming diseased upon exposure was observed and confirmed to have a high degree of heritability in populations of this wild plant species.

## 2 MATERIALS AND METHODS

### 2.1 Study system

*Silene vulgaris* is a short‐lived perennial that is native to Europe, and it has a broad distribution with a weedy, ruderal habit (Keller et al., 2009). Like many species in the Caryophyllaceae, *S. vulgaris* is affected by anther‐smut disease when attacked by fungi in the genus *Microbotryum* (Kido & Hood, 2020). Despite being identified as a smut fungus, *Microbotryum* is more closely related to the rust fungi and is in the basidiomycete subphylum Pucciniomycotina. There is a high degree of host specificity among *Microbotryum* fungi, and the species *M. silenes‐inflatae* is endemic to *S. vulgaris* and its close sister species *Silene uniflora* (Chung et al., 2012; Marsden‐Jones & Turrill, 1957), where the disease is common and results in the same severe reduction in fitness due to complete sterilization of the host through replacement of pollen with fungal spores and the failure of the ovary to mature (Abbate et al., 2018). Infections are systemic (colonizing all or many parts of the host), persistent (infected hosts seldom recover), and the infection is also biotrophic (requiring living host cells for growth). Except upon the initial invasion of the host, partially or weakly diseased plants are rare. The infection status is not discernible until the host produces flowers, when the fungus produces spore‐filled anthers.

### 2.2 Experimental procedures

*Silene vulgaris* seed collections were made from healthy plants in multiple populations in a region bounded approximately by longitudes 44.281–44.395 and latitudes 7.539–7.696 in southern Cuneo Province, Italy (Lerner et al., 2021). Seeds were sampled from natural populations as probably a mixture of full‐ and half‐maternal siblings, that is, from the same dam and one to several sires, and in this study, 12 of these field‐collected seed families were used (collection sites shown in Table 1). The sample of spores (teliospores) of *M. silenes‐inflatae* used in this study also came from this region, from a population of *S. vulgaris* (longitude 44.212, latitude 7.672) that was not among those where the field‐collected families originated, although the degree of local adaptation by the pathogen is unknown. In the life cycle of *Microbotryum*, the spores are diploid, and meiosis occurs upon germination, followed rapidly by a form of selfing (i.e., automixis; Giraud et al., 2008), such that the pathogen is largely homozygous and represents a single lineage used in this study. In order to produce inoculum, this pathogen was maintained in the greenhouse on *S. vulgaris* stock plants for less than three generations, which is unlikely to affect its behavior.

**TABLE 1:** *Silene vulgaris* seed collection localities and family identification.

Family ID	Latitude	Longitude	Genotypes cloned	Ave. clones per genotype	SD in clones per genotype
A	44.195	7.497	17	16.5	1.2
C	44.305	7.668	15	23	3.6
D	44.305	7.668	11	16.9	0.3
E	44.419	7.572	14	24.1	1.5
G	44.274	7.572	17	16.8	0.6
I	44.485	7.661	15	21.3	7.1
T	44.324	7.767	17	16.6	0.7
V	44.371	7.682	17	16.5	1.0
W	44.342	7.617	17	15.8	2.3
3	44.299	7.662	10	23.3	2.9
7	44.340	7.617	10	23.6	4.4
9	44.299	7.662	10	24.9	0.3

Because the outcome of inoculation of a particular plant is binary (healthy or diseased), a vegetative propagation approach was used to quantify the levels of resistance for multiple genotypes within each field‐collected family. The proportion of clones for a particular genotype that become diseased upon inoculation was used as the measure of resistance for each individual genotype. For each of the 12 field‐collected families of *S. vulgaris*, ca. 20 seeds were sown to grow plants, and these were propagated by the rooting of shoot cuttings in perlite with the application of rooting hormone. Vegetative propagation was successful with 170 genotypes, representing each of the collection sites. The number of genotypes (each grown from individual seeds) per field‐collected family for which propagation by cloning was successful ranged from 10 to 17, and the average number of clones per genotype within families ranged from 16 to 23 (Table 1). Overall 3303 clones were produced, randomized for position in the greenhouse, and grown in standard potting mix for several months prior to inoculation with *M. silenes‐inflatae*.

Inoculum for the clones was prepared by agitating ca. 75 diseased flower buds from the diseased stock plants in 1 L of water plus 0.1 mL of surfactant (Triton X‐100), resulting in a spore suspension that was slightly purple colored. A pump sprayer was used to inoculate the cloned plants, applying until runoff twice a week for 6 weeks. Infection was then assessed over the three following months, indicated by the production of flowers with spore‐filled anthers. Because infection of adult plants is an uncommon experimental procedure for this species, on a subset of plants (*n* = 138), the location of the infection was recorded as either being on new shoots from the base of the plant or on a branch of an existing stem.

**FIGURE 2:**
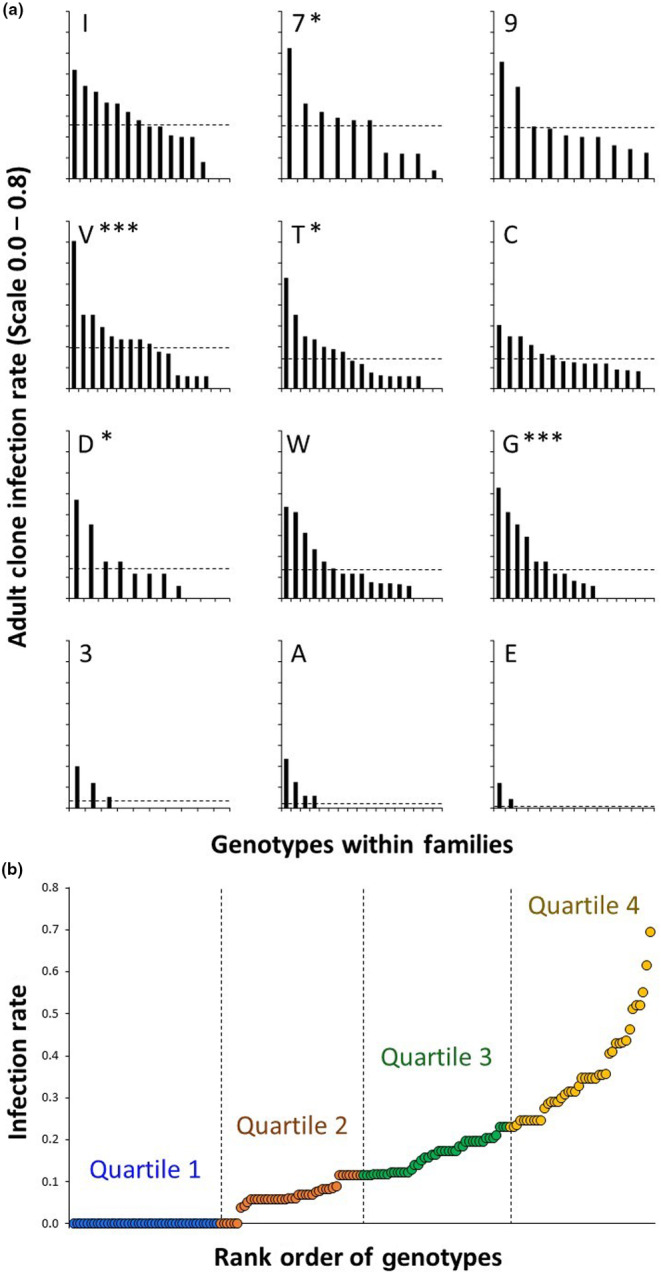
Differences in infection rates of host genotypes of field‐collected *Silene vulgaris* by *Microbotryum silenes‐inflatae*. Infection rate is the proportions that became diseased among clones of a particular genotype; *x*‐axis positions without bars indicate infection rates of zero. (a) Panels represent 12 field‐collected seed families (identified by inset letters), ordered by the greatest to lowest infection rates; genotypes within families are also ordered by the infection rates among their clonal replicates. Dashed horizontal lines indicate family‐level mean infection rates. Asterisks indicate families with significantly high variance in infection rates among the genotypes (Bonferroni‐corrected, *p < .05, **p < .01, ***p < .001). (b) The distribution of infection rates of host genotypes divided into quartiles (demarked by vertical dashed lines).

Because not all clonal replicates of the genotypes became diseased, healthy clones that did not have the sterilized symptoms could be used to produce F1 seeds. The genotypes were ordered according to the infection rates among their clonal replicates and were divided into quartiles (42 or 43 genotypes each) such that Quartile 1 consisted of resistant genotypes with the lowest infection rates, while Quartile 4 consisted of the relatively susceptible genotypes with the highest infection rates (Figure 2). In order to assess the inheritance of the resistance phenotypes, from each resistance quartile, 11 genotypes were randomly chosen to serve as dams and sires for crossing to produce seed offspring as families that were mixtures of full and half‐sib, that is, seeds from the same maternal genotype but where there were multiple sires from within the resistance quartile. An independent second set of 11 genotypes from each quartile was randomly chosen as a replicate of the same crossing procedure. Crossing was performed by rubbing flowers with dehiscent pollen onto mature styles, avoiding self‐pollinating, and seeds from multiple (ca. 10) capsules of the same plant were pooled.

Resistance of the offspring was determined at the family level, as the proportion of inoculated seedlings from the same maternal genotype that became diseased. Seeds were germinated on 0.8% agar containing Murashige and Skoog basal medium at one‐tenth strength and inoculated with *M. silenes‐inflatae* after 10 days with a 4 μL drop of 500 spores/μL of water plus surfactant placed directly on the apical meristem. Two days postinoculation, the seedlings were transplanted to 98‐well Cone‐tainer racks (Stuewe & Sons, Inc.), randomized for position in the greenhouse, and examined for infection status upon flowering.

### 2.3 Data analysis

There are two general approaches to estimating heritability, by the intraclass correlation using the among‐family variance component over total variance or by the regression slope of the offspring phenotype on the mid‐parent phenotype (Falconer, 1986). The former of these is complicated by our sampling of field‐collected families from multiple populations within a broader metapopulation as well as the potential overestimation of among‐family variance due to inbreeding (as detailed below for *S. vulgaris*). Whether field‐collected families differed in the infection rates of their genotypes was assessed by an analysis of variance with nontransformed data. A tentative estimate of the upper limit of heritability was computed using the among‐family variance component and an inbreeding adjustment of dividing the intraclass correlation, *t*, by [1 + *F*_IS_ (1−*t*)], where F_IS_ is the inbreeding coefficient (Nyquist & Baker, 1991), and then multiplying this by between two and four due to the possible range of full‐sib to half‐sib relationship of offspring within the field‐collected families (Falconer, 1986). Inbreeding coefficients in *S. vulgaris* have been estimated at 0.178 in McCauley (1998), 0.353 in Glaettli et al. (2006), and 0.024 in Kahl et al. (2021), and an average of these values (0.185) was used in the adjustment.

To test whether infection rates of the genotypes varied significantly within the field‐collected plant families and did not reflect only variation due to probabilistic infection and a finite number of clonal replicates, a simulation approach was used. Using the number of clones scored per genotype and assuming an infection probability based on the weighted average infection rate among genotypes within the field‐collected family, 10,000 replicates of simulated infections were used for calculating the within‐family variances. The observed within‐family variance was compared with the distribution of simulated variances assuming no differences among genotypes within families for infection probabilities, and the statistical threshold for exceeding standard alpha values was corrected by the Bonferroni method for 12 independent tests. The modality of the distribution for individual genotype‐level infection rates was assessed using the bimodality coefficient on the arcsine‐square‐root‐transformed proportional data (“mousetrap” package in R; Kieslich & Henninger, 2017), with values above 0.556 indicating a bimodal distribution (Pfister et al., 2013).

Linear regression with quartile and replicate as predictor variables for offspring infection rates was used to test whether offspring from the different resistance quartiles varied in infection rates. This analysis showed no significant effect of replicate, and therefore, the data from the two replicates were combined. Pairwise *Z*‐tests were used to determine the differences in infection rates among offspring produced from the four resistance quartiles, with a Bonferroni correction of the critical threshold for significance at alpha = 0.05 due to multiple tests.

To assess the relationship between the offspring infection rates and the parental infection rates, the parental infection rates were standardized due to the difference in inoculation methods with an expected difference in seedling and adult overall susceptibility (Bruns et al., 2022); the effect of this transformation is to allow the standard interpretation of the slope of this relationship but does not affect the statistical significance. Thus, the adult infection rates were multiplied by the overall seedling infection rates divided by overall adult infection rates. The mid‐parent value was then calculated as the average between the adjusted infection rate of the maternal genotype and average infection rate of adult genotypes within a resistance quartile. We used least squares regression, weighted by the number of clonal replicates, on arcsine‐square‐root‐transformed proportional data, to determine the slope of the relationship between the parents' infection rates and offspring infection rates. We performed a weighted Pearson correlation test on arcsine‐square‐root‐transformed proportional data to assess the significance of the correlation. To test whether the variation in resistance among genotypes within field‐collected families affected the offspring infection rates, we used Pearson correlation test to compare the adjusted maternal genotype's deviation from the within‐family mean infection rate to the deviation of the maternal genotype's offspring from their within‐family mean infection rate; this correlation used transformed proportional data and was weighted by the number of parental genotypes used per family. Statistical analyses were conducted in SPSS version 27.

## 3 RESULTS

### 3.1 Infection of clones of genotypes from field‐collected families

Among 3303 clones of adult *S. vulgaris* plants, 493 (15%) became diseased following inoculation with *M. silenes‐inflatae*. With regard to the site of infection on adult plants, about half of the infections occurred on new shoots originating from the base of the plant and half occurred on a branch originating from the node of an existing stem; among 138 diseased plants assessed for infection location (in families I, C and E), 59 had infections on new shoots from the base of the plant, 58 had infections on a branch of an existing stem, and 21 had infections originating at both locations.

For the 170 plant genotypes, infection rates (the proportion of clonal replicates of a particular genotype that became diseased) ranged from 0 to 0.71. The modality of this individual genotype‐level trait distribution was unimodal (bimodality coefficient = 0.454, critical value for indicating bimodality is 0.556). Infection rates varied significantly among the 12 field‐collected families (dashed lines in Figure 2; *F*_11,158_ = 5.905, *p* < .001). The among‐family variance component in the infection rates of genotypes was 0.006, with a total variance of 0.022. Correcting for inbreeding and the relationship among siblings (see Section 2), the range of intraclass correlation estimates of heritability for the disease resistance was from 0.464 to 0.928.

Infection rates also varied significantly among genotypes within families that could reflect trait segregation of allelic variation or the contributions from differing sires. The among‐genotype variance within five families was significantly greater than produced by simulated infection occurrences based on the family average infection rate and the number of clonal replicates, with two families having significant variances at the *p* < .001 level and three families having significant variances at the *p* < .05 level (Figure 2a). The strongest example of this was in family V, with a family‐level infection rate of 0.20 but one genotype having an infection rate of 0.71 among 16 clones, and family G, with a family‐level infection rate of 0.14 and genotypes ranging from infection rates of 0.53 to 0.00. For field‐collected families that gave no evidence of infection rate differences among genotypes, there were genotypes within most susceptible families where no clones became diseased (e.g., family I), and there were genotypes within the most resistant families where at least some clones became diseased (e.g., family E), suggesting that infection by anther smut is probabilistic rather than deterministic based on a given genotype (noting that all inoculated hosts were exposed to large number of pathogen spores).

### 3.2 Infection of offspring seedlings

**FIGURE 3:**
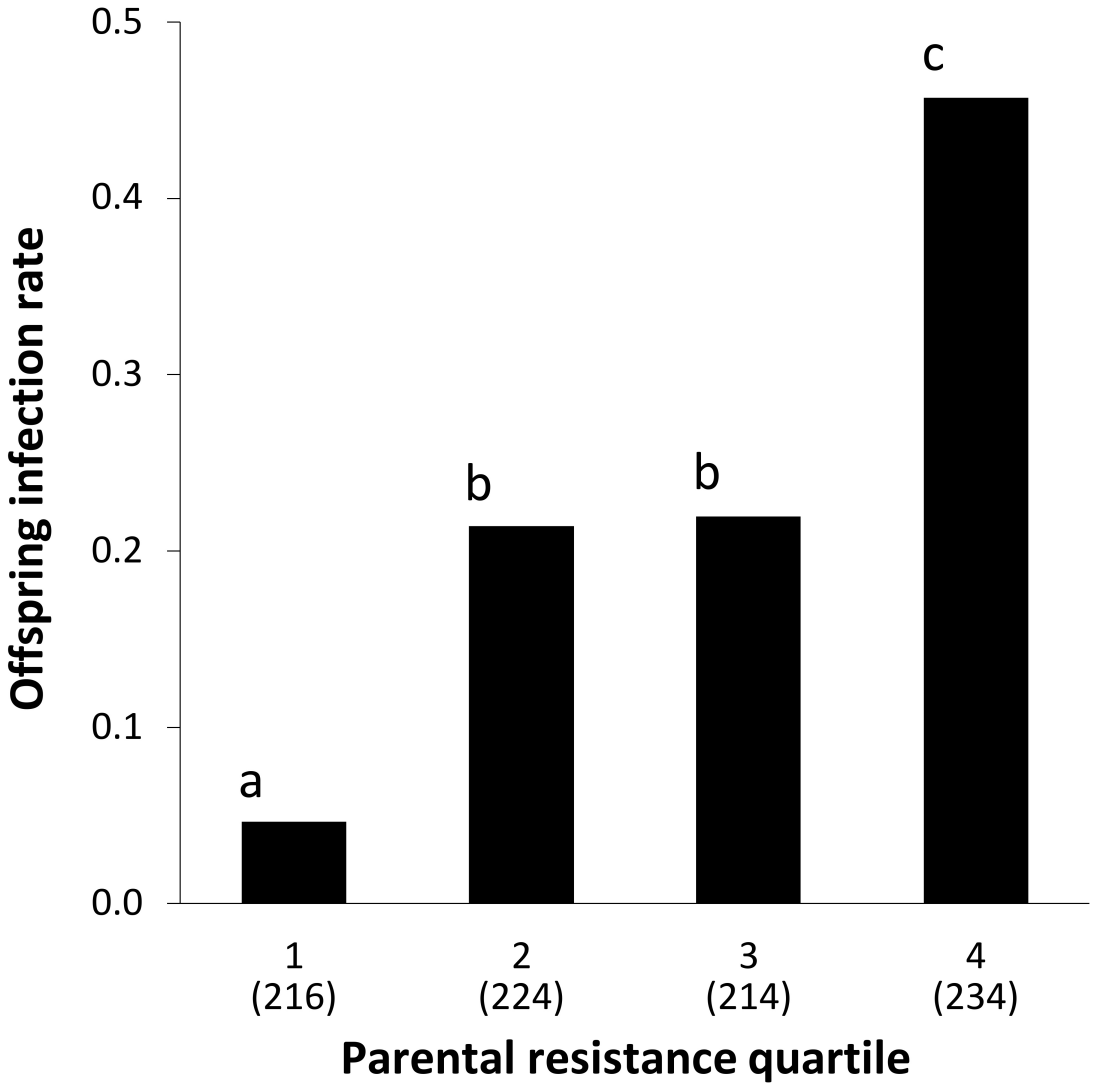
Infection rates of *Silene vulgaris* by *Microbotryum silenes‐inflatae* for inoculated offspring seedlings derived from crossing among parental genotypes grouped according to infection rates of their adult‐inoculated clones. Quartiles 1 through 4 reflect the parental resistance levels from the lowest to the highest infection rates, respectively. Infection rate is the proportion diseased among offspring, and the parenthetical numbers below the quartiles indicate sample sizes of inoculated seedlings. Differing letters indicate parental quartiles with statistically significant differences in offspring infection rates (Bonferroni‐corrected alpha of 0.001). *Z*‐scores for Quartiles 1 versus 2 = −5.207; 1 versus 3 = −5.3; 1 versus 4 = −9.93; 2 versus 3 = −0.136; 2 versus 4 = −5.493; 3 versus 4 = −5.29.

The grouping of genotypes into resistance quartiles based on the adult infection rates among their clones (Figure 2b) was then used for crossing within the quartiles to generate half‐sib offspring, the infection rates of which were assessed for inheritance of the resistance trait. Among 888 seedling‐inoculated offspring plants, 209 (24%) became diseased with *M. silenes‐inflatae*, consistent with a known greater seedling susceptibility (see Bruns et al., 2022). For the 88 offspring families, infection rates based on the proportion of seedlings that became diseased ranged from 0 to 0.75 (average number of seedlings inoculated per family = 10.0). From the linear regression analysis including the four quartiles and the two replicates of selected parental groupings with resistance quartiles as predictors, the parental quartiles significantly correlated with the infection rate of the offspring seedlings, but the replicates had no significant effect (Wald $\chi_{\left(3\right)}^{2}$ for quartile = 93.36, *p* < .001, Wald $\chi_{\left(1\right)}^{2}$ for replicate = 0.067, *p* = .796), and the replicates were combined for further analyses. The Bonferroni‐corrected pairwise *Z*‐tests between the seedling infection rates indicated that the offspring from parents in the most resistant parental quartile (Quartile 1) had a significantly lower infection rate than offspring from the other quartiles, while the offspring from the most susceptible parental quartile (Quartile 4) had a significantly higher infection rate (Figure 3). The two intermediate quartiles (2 and 3) had moderate infection rates, did not differ from each other significantly, and were nearly identical.

**FIGURE 4:**
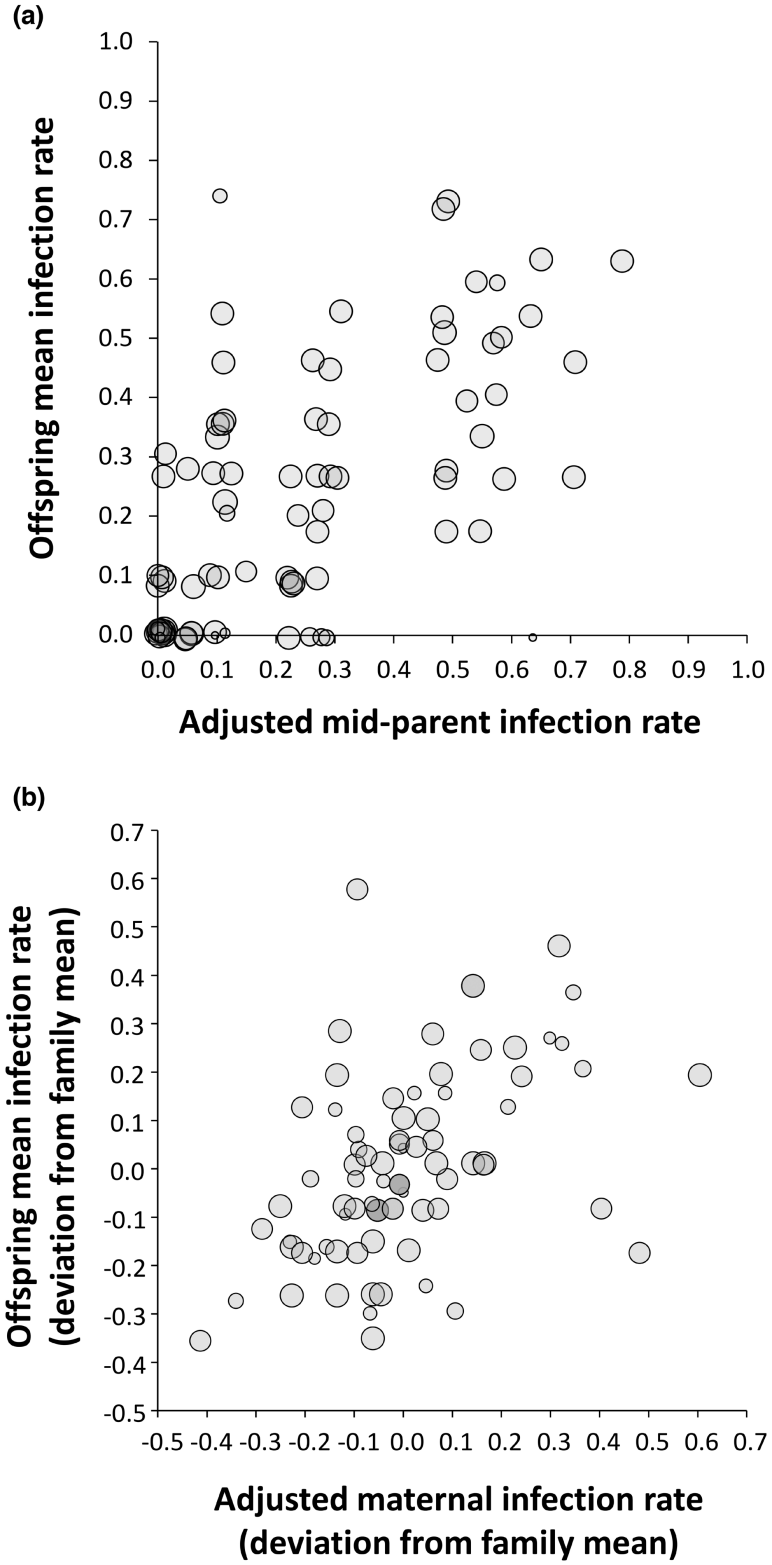
Relationships between parent and offspring resistance in *Silene vulgaris* to infection by *Microbotryum silenes‐inflatae*. (a) The relationship between the mid‐parent infection rate (adult inoculation) and offspring infection rate (seedling inoculation). The mid‐parent infection rate was adjusted by the product of overall seedling/adult infection rates, standardizing the mean and variance. Points shown were jittered randomly to minimize overlap. Circle diameters reflect sample size as the number of clonal replicates per genotype, and the line represents a weighted least squares regression. (b) The effect of variation within field‐collected families on the infection rate of offspring. The *x*‐axis shows the maternal genotype's deviation from the within‐family mean infection rate (adult inoculation), and the *y*‐axis shows the deviation of the maternal genotype's offspring from the within‐family mean infection rate (seedling inoculation); maternal infection rates adjusted to standardize the variances. Circle diameters reflect the number of maternal genotypes per family, and the line represents weighted least squares regression.

Despite variation among offspring mean infection rates possibly influence by the small sample size per offspring group, there was a significant relationship between the mid‐parent infection rate of the cloned adults and the seedling infection rate in their offspring (proportional data transformed, weighted Pearson *r* = .705, *p* < .001); because of the difference in inoculation methods between the parent and offspring groups, the adult infection rates used for the mid‐parent comparison were adjusted by the product of overall seedling/adult infection rates to standardize the means and variance. The weighted regression of transformed proportional data for the offspring infection rate on the adjusted mid‐parent infection rate showed a slope of 0.701 (SE = 0.024; Figure 4a).

Resistance variation that appeared within the 12 field‐collected families (Figure 2 above) also affected the offspring infection rate (Figure 4b). The maternal genotypes' deviation from the within‐family mean infection rate was significantly correlated with their offsprings' deviation from the within‐family mean infection rate for offspring from the same field‐collected family (weighted Pearson *r* = .454, *p* < .001). Weighted regression of the deviations from the within‐family means for the offspring on the maternal genotypes showed a slope of 0.489 (SE = 0.034).

## 4 DISCUSSION

There was a high degree of resistance variation to anther‐smut disease in *S. vulgaris* collected from natural populations, where using hosts for crossing based on ranked phenotypes provided strong evidence of heritability through its effect on the resistance values in the offspring. Because there is a binary outcome for a particular host plant (healthy or diseased) following exposure to anther smut, the assessment of infection among a set of clones of host genotypes is critical for understanding individual variation in the resistance phenotype. Where most studies on anther smut assess differences in family‐level resistance (i.e., infection rate among siblings; Bruns et al., 2022; Carlsson‐Granér, 1997; Carlsson‐Granér & Thrall, 2015; Chung et al., 2012; Kaltz & Shykoff, 2002; Lerner et al., 2021), such variation could reflect either differing segregation ratios of strongly deterministic susceptible versus resistant phenotypes or more continuous variation in the probability of infection, the latter often assumed to reflect the contribution from numerous quantitative trait loci. In the current study addressing the individual host genotype level, infection of *S. vulgaris* upon exposure to its endemic pathogen, *M. silenes‐inflatae*, appears to be probabilistic, with infection rate being a continuously variable trait of a broad unimodal distribution. It is not known whether repeated inoculations would lead the *M. silenes‐inflatae* to overcome such resistance. While quantitative disease resistance is generally considered to lessen the intensity of infections rather than to prevent infection (French et al., 2016), there are other similar examples where continuously varying resistance traits can affect probabilities of being affected by disease (i.e., threshold traits) and not just its intensity, such as the development of physical and chemical barriers or the degree of matching between multiple host and pathogen recognition loci (Buckingham & Ashby, 2022; Price et al., 2004).

A prior methodological study using the tissue culture propagation of *S. vulgaris* genotypes in order to quantify resistance drew a contrasting conclusion and found discrete categories of highly resistant or susceptible genotypes segregating within full‐sib host families (Cafuir et al., 2007), suggesting a relatively simple genetic control over the disease resistance. However, that study used a foreign pathogen, *M. lychnidis‐dioicae* from *S. latifolia*, to which *S. vulgaris* had been exposed as a localized host shift (Antonovics et al., 2002). The possibility that different mechanisms govern resistance to endemic and foreign *Microbotryum* pathogens is supported by the Lerner et al. (2021) study, which found a number of *S. vulgaris* outlier families that deviated significantly from the otherwise positive correlation of family‐level infection rates by these same two pathogen species. In comparison with Cafuir et al. (2007), the current study found a relatively small but still significant degree of resistance variation within the field‐collected families. This was seen in the cases of higher among‐genotype variances within families than expected by chance alone, as well as in the correlation between deviations from the within‐family means for maternal parent and offspring infection rates. It is unclear whether such variation among siblings is due to the presence of some major‐effect loci co‐occurring within the broader quantitative variation or, perhaps more likely, the influence of different seeds within field‐collected families having separate sires. Further studies on the paternity patterns within field‐collected families or the use of full‐sib crossing designs could help resolve this uncertainty.

Both our analysis of variance and offspring–parent regression indicate that resistance in *S. vulgaris* to infection by *M. silenes‐inflatae* is highly heritable, but a precise measure of narrow‐sense heritability remains challenging. The ability of trait values of plants grown under greenhouse conditions from field‐collected seeds to predict the resistance level of their offspring indicates that maternal effects from environmental heterogeneity in the field were not a primary determinant of the observed individual variation. The use of clones produced by vegetative cuttings necessitates the measuring of resistance at the adult stage for the parental generation, where infection rates are markedly lower than for the seedling stage used for the offspring inoculations. The clones that served as parental plants indeed had a lower overall infection rate even though they received more doses of inoculum. This decrease in susceptibility with age is a general phenomenon and occurs across host species of anther‐smut disease (Bruns et al., 2022). As a result, adjustment of the adult infection rate was used to standardize the statistical comparison with the seedling‐inoculated offspring, but these complications mean that the slope of the offspring–mid‐parent regression is likely not a precise indicator of narrow‐sense heritability. The use of the intraclass correlation in the current study is also imprecise due to the parental families not coming from the same local population, uncertainty of sibling relationships, and, especially in the field, natural rates of inbreeding vary markedly among *S. vulgaris* populations (Glaettli et al., 2006; Kahl et al., 2021; McCauley, 1998) with potential effects on intraclass correlation (Nyquist & Baker, 1991). However, our use of different aged plants for the treatment of the offspring and parent generations with the same type of inoculum was likely not a serious limitation to interpreting the resistance variation. We have previously shown that infection rates of *S. vulgaris* at seedling and adult stages of the same field‐collected generation were positively correlated (Bruns et al., 2022). This indicates that using seedling inoculations in *S. vulgaris*, which are much simpler and quicker to work with than adult inoculations, is relevant to how resistance affects disease transmission in the field either through infections of seedlings or adults. This consistency of resistance patterns across ages in *S. vulgaris* is in contrast to patterns seen in other hosts of anther‐smut disease (Bruns et al., 2022), where the mechanisms of disease resistance can evolve independently at the adult and seedling stages. Compared with other hosts of anther‐smut disease, the efficacy of resistance evolution in *S. vulgaris* may be enhanced because selection acting at one age would produce a correlated response at the other age.

While we have shown that there is quantitative inheritance of resistance in *S. vulgaris*, additional considerations about the general qualitative versus quantitative dichotomy require further consideration. While there can be exceptions, it is often assumed that qualitative resistance shows specificity of interactions with different pathogen genotypes, contrasted by a lack of specificity in quantitative resistance (Kushalappa et al., 2016; Poland et al., 2009). However, there is evidence of pathogen genotype specificity in the interactions with some other hosts of anther smut (e.g., *S. latifolia* and *Dianthus carthusianorum*). While we cannot draw a conclusion about *M. silenes‐inflatae* here, on those other anther‐smut diseases there is often maladaptation of the *Microbotryum* species for infecting plants from sympatric compared with allopatric host populations (Kaltz et al., 1999; Koupilová et al., 2021), which indicates a degree of host genotype‐by‐pathogen genotype interaction. Moreover, theoretical study suggests such parasite local maladaptation is particularly unlikely where the host–pathogen coevolution is governed only by quantitatively inherited resistance (Ridenhour & Nuismer, 2007). Here, we used a single strain of *M. silenes‐inflatae* for the inoculations of *S. vulgaris*, precluding a direct test of such resistance specificity; however, Lerner et al. (2021) found that infection rates among a subset of the same *S. vulgaris* families were consistently replicated when using with a second strain of *M. silenes‐inflatae*. Because a single pathogen lineage was used here, a broader testing of pathogen genotypes is warranted, and there remains the possibility of resistance specificity in *S. vulgaris*, even within the observed quantitative resistance variation. For instance, several plant–pathogen systems are described as having strain specificity for quantitative resistance (so‐called, minor gene‐for‐minor gene interactions; Langlands‐Perry et al., 2021; Niks et al., 2015; Poland et al., 2009), and there can be quantitative variation that modulates the strength of more traditional qualitative gene‐for‐gene responses, like the hypersensitive reaction (French et al., 2016; Penning et al., 2004; Sun et al., 2004).

This study was motivated by prior work revealing a range of family‐level infection rates with anther‐smut disease and the uncertainty of the underlying mode of resistance inheritance. The highly significant heritability of resistance in *S. vulgaris* that we demonstrated, combined with the severe fitness consequences of infection by the anther‐smut fungi, predicts a strong evolutionary response in the host phenotype under high disease pressure (Bruns et al., 2015, 2019). However, the quantitative nature of resistance variation to this endemic pathogen, if determined by multiple loci, should result in less dynamic allele frequency changes than if resistance were primarily determined by very few major‐effect loci, even though phenotypic response to polygenic selection can still be rapid (Stephan, 2021). It remains a possibility for further investigations to determine whether quantitative resistance variation in this endemic disease system exists in a mixture with significant qualitative resistance and pathogen strain specificity, as is suggested to occur in natural plant–pathogen interactions generally (Burdon & Thrall, 2009; Laine et al., 2011).

## AUTHOR CONTRIBUTIONS

**Michael E. Hood:** Conceptualization (equal); data curation (equal); formal analysis (equal); funding acquisition (equal); investigation (equal); methodology (equal); project administration (equal); writing – original draft (equal); writing – review and editing (equal). **Sydney Nelson:** Conceptualization (equal); data curation (equal); investigation (equal); writing – review and editing (equal). **Jae‐Hoon Cho:** Data curation (equal); investigation (equal); methodology (equal); writing – review and editing (equal). **Michelle Launi:** Formal analysis (equal); investigation (equal); writing – review and editing (equal). **Janis Antonovics:** Conceptualization (equal); funding acquisition (equal); writing – review and editing (equal). **Emily L. Bruns:** Conceptualization (equal); funding acquisition (equal); writing – review and editing (equal).

## CONFLICT OF INTEREST STATEMENT

The authors declare no competing interests.

## Data Availability

The data that support the findings of this study are openly available in Dryad at https://doi.org/10.5061/dryad.fj6q57426.
